# Assessment of Drinking Water Sold from Private Sector Kiosks in Post-Earthquake Port-au-Prince, Haiti

**DOI:** 10.4269/ajtmh.16-0692

**Published:** 2017-10-18

**Authors:** Molly Patrick, Maria Steenland, Amber Dismer, Jocelyne Pierre-Louis, Jennifer L. Murphy, Amy Kahler, Bonnie Mull, Melissa D. Etheart, Emmanuel Rossignol, Jacques Boncy, Vincent Hill, Thomas Handzel

**Affiliations:** 1 Division of Global Health Protection, Center for Global Health, Centers for Disease Control and Prevention Atlanta, Georgia;; 2 Division of Foodborne, Waterborne and Environmental Diseases, National Center for Emerging and Zoonotic Infectious Diseases, Centers for Disease Control and Prevention, Atlanta, Georgia;; 3 Haitian Ministry of Public Health and Population (MSPP), Port-au-Prince, Haiti

## Abstract

Consumption of drinking water from private vendors has increased considerably in Port-au-Prince, Haiti, in recent decades. A major type of vendor is private kiosks, advertising reverse osmosis-treated water for sale by volume. To describe the scale and geographical distribution of private kiosks in metropolitan Port-au-Prince, an inventory of private kiosks was conducted from July to August 2013. Coordinates of kiosks were recorded with global positioning system units and a brief questionnaire was administered with the operator to document key kiosk characteristics. To assess the quality of water originating from private kiosks, water quality analyses were also conducted on a sample of those inventoried as well as from the major provider company sites. The parameters tested were *Escherichia coli*, free chlorine residual, pH, turbidity, and total dissolved solids. More than 1,300 kiosks were inventoried, the majority of which were franchises of four large provider companies. Approximately half of kiosks reported opening within 12 months of the date of the inventory. The kiosk treatment chain and sales price was consistent among a majority of the kiosks. Of the 757 kiosks sampled for water quality, 90.9% of samples met World Health Organization (WHO) microbiological guideline at the point of sale for nondetectable *E. coli* in a 100-mL sample. Of the eight provider company sites tested, all samples met the WHO microbiological guideline. Because of the increasing role of the private sector in drinking water provision in Port-au-Prince and elsewhere in Haiti, this assessment was an important first step for government regulation of this sector.

## INTRODUCTION

In metropolitan Port-au-Prince, Haiti, the types of drinking water sources used have changed considerably in recent decades. The proportion using piped water sources has decreased, whereas the proportion using drinking water sources provided by the private sector has increased. At the time of the last Demographic and Health Survey (DHS) in 2012, 16.4% and 21.1% of households in metropolitan Port-au-Prince reportedly used piped water in the home and from a public tap stand for drinking water, respectively, compared with 23.5% and 64.5% from the DHS in 2000.^1,2^ Meanwhile, the use of bottled water for drinking increased substantially over the same period. The DHS in 2000 reported that 2.1% of households used bottled water as their primary source of drinking water, whereas in 2012 it was reported that 49.0% of households used this source type.^1,2^ There is evidence of private sector drinking water sources becoming established in other parts of the country as well.^2–4^

The “bottled water” category includes several sources, including bottled water, sachet water (water sold in sealed plastic bags), and private kiosk water. The private-sector kiosks generally operate as franchises of provider companies and advertise reverse osmosis (RO) membrane filtration–treated water for sale by one or five gallon volumes. The proportion of the population using private kiosks is unknown; however, the growth of the sector has been substantial since the earthquake and cholera outbreak of 2010. In fact, a household survey conducted at the outset of the cholera outbreak in vulnerable neighborhoods in metropolitan Port-au-Prince found that reported use of private kiosks for drinking water had almost doubled since the onset of the outbreak; even in these resource-limited areas, almost half (47.6%) of survey respondents reported using private kiosks at the time of the survey in December of 2010.^5^ However, there was no information available on the quality of water sold from these private kiosks.

Given the increasing importance of private kiosks in Port-au-Prince, and more broadly the private sector, in drinking water provision, we conducted a cross-sectional assessment to describe the private kiosk sector in metropolitan Port-au-Prince in terms of 1) scale and geographical distribution of private kiosks; 2) major provider companies and supply and treatment chain; 3) sales volume and price; and, most importantly, 4) water quality at point of sale and site of production. This project was a collaborative effort with other Haitian government entities including the National Directorate for Drinking Water and Sanitation (DINEPA) and the Ministry of Commerce and Industry (MCI).

## METHODS

The kiosk assessment was conducted in the six communes of metropolitan Port-au-Prince (Tabarre, Cité Soleil, Delmas, Port-au-Prince, Carrefour, and Pétion-ville) over a 6-week period during July and August, 2013. The goal was to cover all of the communes; however, urban areas with higher population density were preferentially chosen when only a portion of a commune could be covered due to time constraints. The assessment included both an inventory of kiosks and water quality testing on a sample of those inventoried. On completion of the kiosk inventory, 10% of the area covered was randomly selected and revisited to validate the completeness of the inventory. After preliminary analyses of inventory data, the production sites of the major provider companies were visited and a water sample was taken. The field teams, comprised of eight Ministry of Public Health and Population (MSPP) Sanitary Agents and two hired supervisors, were trained on assessment procedures over 3 days.

### Kiosk inventory.

ArcGIS 10.1 (Esri, Redlands, CA) was used to divide the six communes into approximately 2 km^2^ segments using Open Street Map (© OpenStreetMap contributors) road data as boundaries.^6,7^ Eight 3-person teams consisting of an MSPP Sanitary Agent, motorcycle driver, and local guide were given segment maps, Garmin 62stc Global Positioning System (GPS) units (Garmin International, Inc., Olathe, KS) and were instructed to traverse all roads that could be accessed by a water truck using the continuous path of travel method.^8–10^

Private kiosks were defined as vendors located in a commercial building advertising RO-treated water for sale. Kiosks that were closed at the time of the visit were recorded with GPS coordinates and a photo taken. These kiosks were excluded from the inventory and subsequent analyses as it could not be established if the kiosks were temporarily or permanently closed (i.e., abandoned). At each open private kiosk, team members explained the purpose of the inventory to the operator, obtained informed consent to conduct a short questionnaire, tested free chlorine residual on-site and requested permission to collect a water sample (at select kiosks, described in the Water Quality Testing section). A standardized questionnaire was used to collect kiosk name, date of opening, provider company name, water delivery mechanisms, kiosk equipment, sales volume, and sale price. The owner name and contact information, kiosk street address, GPS coordinates, and a photo were also taken at each kiosk.

### Kiosk inventory validation.

From the full list of segments included in the inventory, 10% were randomly selected and reinventoried by the two team supervisors to validate/evaluate the completeness of the kiosk data. Using the same continuous path of travel method, supervisors recorded GPS coordinates and a photo of each private kiosk located.

### Provider company site visits.

Data from the inventory were used to identify the major provider companies and site visits were conducted at seven of the eight largest companies. This visit was prearranged using contact information from a registry of companies provided by MCI. After informed consent was obtained, the GPS coordinates were taken and a questionnaire was used to determine water source, treatment methods, and transport mechanism to kiosks. With permission, water samples of raw, treated and tanker truck (if available) water were also taken.

### Water quality testing.

At every kiosk, a test for free chlorine residual was conducted on-site using a Pocket Colorimeter II (Hach Company, Loveland, CO). For the microbiological and physicochemical samples, a systematic sampling strategy was used by the teams such that the first three kiosks encountered in the morning and the first two in the afternoon (five kiosks per day per team) were sampled. At each of these kiosks, the tap was decontaminated using a sterile ethanol wipe and two water samples were collected using aseptic techniques. One 100-mL sample was collected in a sterile, 125-mL Nalgene bottle for select physicochemical analyses, and one 100-mL sample was collected in a sterile IDEXX sample bottle containing sodium thiosulfate for microbiological analysis. Samples were stored in cooler boxes with ice packs and delivered by the two supervisors twice daily to the MSPP National Public Health Laboratory (LNSP) within 4 hours of collection. On arrival at LNSP, samples were tested by Centers for Disease Control and Prevention (CDC) staff for pH and total dissolved solids (TDS) using a portable conductivity/TDS/pH meter (Hanna Instruments; Woonsocket, RI) and for turbidity using a portable turbidimeter (Model 2100P; Hach Company). Tests for *Escherichia coli* were conducted using Colilert-18 media with the IDEXX Quantitray/2000 system (IDEXX Laboratories, Westbrook, ME). Kiosks with samples that tested positive for *E. coli* were resampled by the two supervisors as soon as possible within the assessment period. Kiosk operators were informed of the positive result and reason for the retest. Negative controls were included in the cooler boxes daily for quality control during transport, and both positive and negative microbiological controls were also assayed in the laboratory each day.

At the provider company sites, water was tested on-site for free chlorine residual and two 100-mL samples were collected for chemical and microbiological analyses at LNSP, using the same procedures and parameters tested at the kiosk level.

### Data analysis.

During the assessment period, two data entry technicians entered data daily in an Epi Info 7 database (CDC; Atlanta, GA). Descriptive and statistical analysis of inventory and water quality data was conducted using SAS, version 9.3 (SAS Corporation; Cary, NC). χ^2^ was used for comparative analyses, with significance reported at the 0.05 level and χ^2^ test of equivalence of proportions used to compare *E. coli* results of companies to the overall proportion. ArcGIS 10.1 and the Haitian Institute of Statistics and Information’s 2011 projections of population per census tract were used to estimate the population of the segments covered by the inventory. Census tract projections were based on the 2003 census data and a rapid assessment conducted after the earthquake.^11^ First, the percent of the census tract and area located within each inventory segment was calculated. Next, the population of the census tract was attributed proportionally to the area inside the segment, with the final population per segment the sum of all these proportional segment populations. ArcGIS 10.1 was also used to compare the data from the kiosk inventory validation step to the original data.

Supplemental analyses on cost of using private kiosk water were conducted based on World Health Organization (WHO) estimates of daily water needs for drinking only (3L per capita per day) and basic consumption (drinking and food preparation; 7.5L per capita per day),^12^ using the per capita gross national income (GNI) at the time of the inventory (USD800.00).^13^ Water quality data were analyzed with respect to WHO Drinking Water Quality Guidelines.^14^ The physicochemical parameters do not have health-based target guidelines. The target aesthetic or normal ranges are 6.5–8.5 for pH (“acceptable range”), less than 1 Nephelometric Turbidity Units (NTUs) for turbidity (a measure of suspended solids and sediment), and less than 600 mg/L for TDS (higher than 1,000 mg/L water becomes unpalatable). For the *E. coli* analyses, the health-based WHO guideline states that there should be no *E. coli* detectable per 100-mL sample. For the standard method used, this is measured as < 1 most probable number (MPN) per 100 mL. The *E. coli* results are presented according to WHO stipulated levels of health risk corresponding to ranges of *E. coli* contamination.^14^ *Escherichia coli* results were stratified by provider company, delivery mechanism, and treatment mechanism on-site to look for associations with contamination.

The protocol was approved by the National Bioethics Committee of Haiti’s Ministry of Public Health and Sanitation. The protocol was also reviewed by the CDC and determined to be a nonresearch public health program activity.

## RESULTS

### Kiosk inventory.

**Figure 1.**
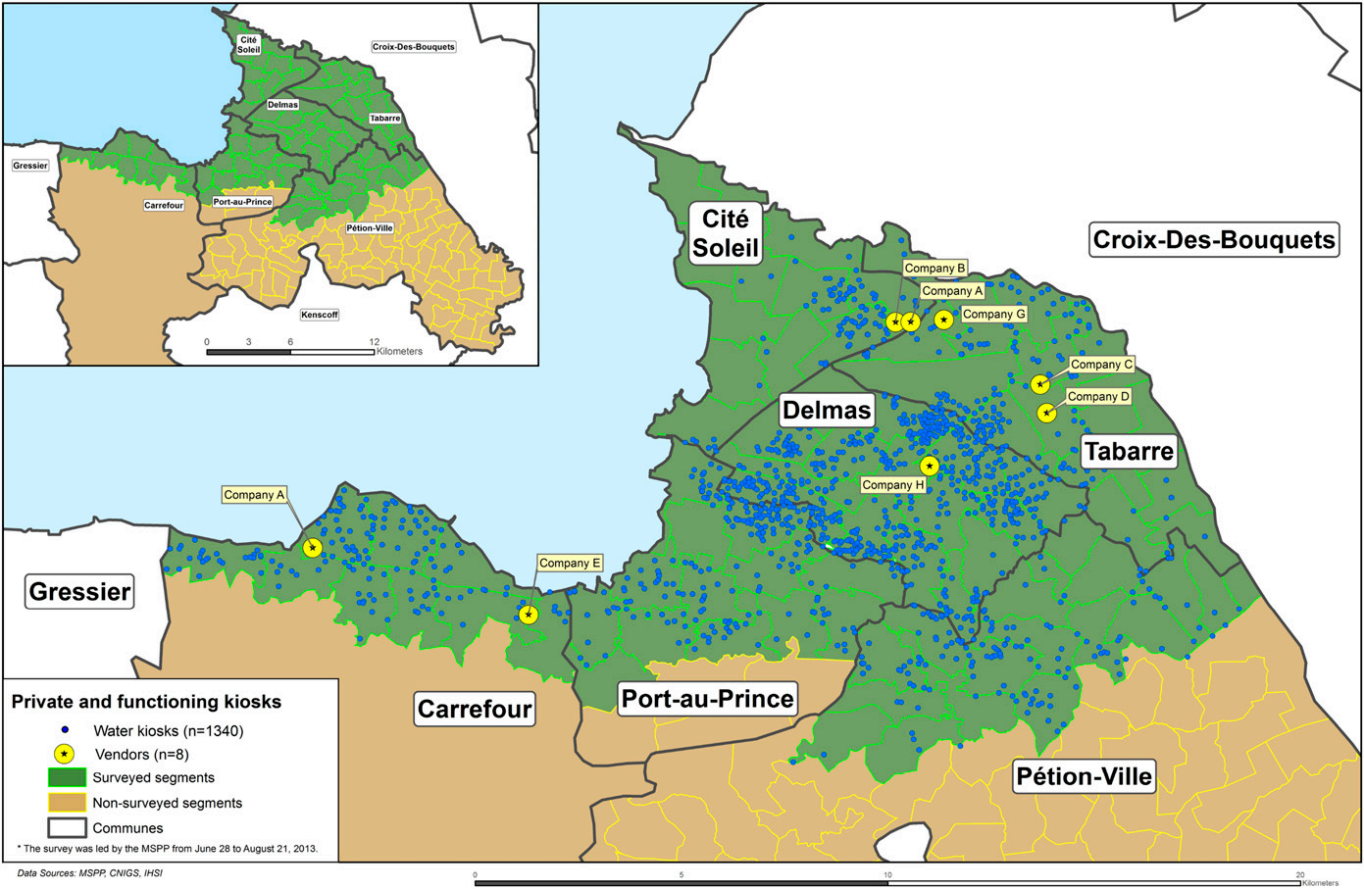
Full land area targeted by the kiosk inventory in metropolitan Port-au-Prince shown in dark and light grey in top left inset. Covered areas shown in dark grey, including location of kiosks and provider company sites visited. This figure appears in color at www.ajtmh.org.

In total, the population covered by the inventory was approximately 1.89 million persons or 88.2% of the 2013 population of metropolitan Port-au-Prince. Of the six communes of metropolitan Port-au-Prince, Tabarre, Cité Soleil, and Delmas communes were fully covered. Only partial coverage was obtained in Port-au-Prince (65.4% covered), Pétion-ville (18.3% covered), and Carrefour (11.4% covered) communes due to time constraints (Figure 1; upper left inset).

### Scale and distribution.

A total of 1,340 open kiosks were identified in the six communes. An additional 153 kiosks were located (either temporarily or permanently closed) at the time of the inventory; however, these are excluded from the analyses. Delmas commune contained more than 40% of the kiosks inventoried, followed by Port-au-Prince commune (Table 1).

**Table 1:** Number of kiosks, kiosk per population and geographical unit, and population density per commune covered by the inventory in metropolitan Port-au-Prince, July–August 2013

Commune (*N* = 1,340)	No. of kiosks (%)	Kiosks/100,000 persons	Population density (pop/km^2^)	Kiosks/km^2^
Delmas	561 (41.9)	128.8	14,988	19.3
Port-au-Prince*	211 (15.7)	38.9	21,652	8.3
Tabarre	181 (13.5)	116.7	4,885	5.7
Carrefour*	159 (11.9)	51.5	14,760	7.6
Pétion-ville*	141 (10.5)	68.4	6,429	4.4
Cité Soleil	87 (6.5)	37.3	9,664	3.6

* Only partial coverage obtained; area and population density represent only the areas covered.

Of the total area covered by the inventory, Delmas had twice as many kiosks per population unit than the other areas, closely followed by Tabarre. Notably, this did not mirror the overall population density of the areas inventoried. The portion of Port-au-Prince covered had the highest population density and the second fewest number of kiosks per population unit. The density of kiosks per land area was highest in Delmas with 19.3 kiosks per km^2^ and lowest in Cité Soleil with 3.6 kiosks per km^2^.

**Figure 2.**
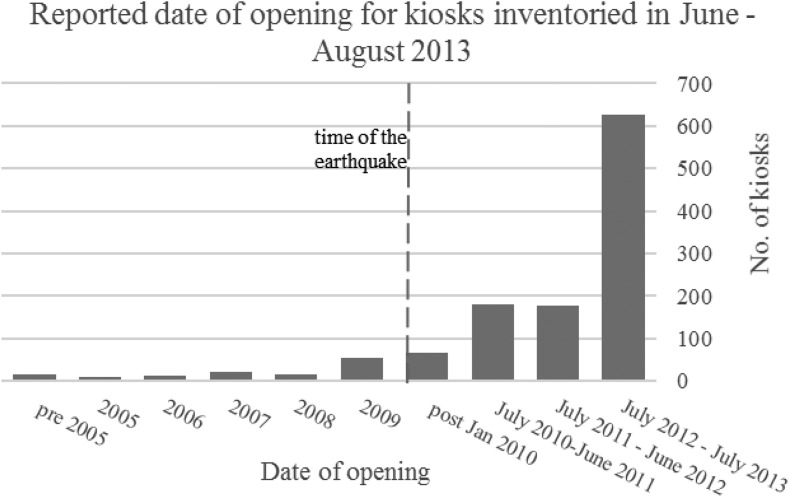
Reported year of opening for kiosks inventoried in metropolitan Port-au-Prince, July–August 2013.

The majority of respondents (83.7%) reported that the kiosk had been open for 3 or fewer years (Figure 2). Just more than 600 respondents (53.3%) reported that the kiosk opened within the year prior to the inventory.

### Provider companies and kiosk specifications.

Of the 1,266 kiosks (94.5%) where a response was provided for the provider company name on the questionnaire, a total of 67 different providers were recorded. However, the top four companies accounted for more than 80% of the kiosks inventoried (Table 2). The majority of these kiosks (91.0%) were franchises of the eight companies listed in Table 2. Company A, accounted for just under half of the total number of kiosks included in the inventory.

**Table 2:** Number of kiosks inventoried per provider company in metropolitan Port-au-Prince, July–August 2013

Provider company (*N* = 1266)	No. of kiosks (%)
A	588 (46.4)
B	218 (17.2)
C	150 (11.9)
D	72 (5.7)
E	45 (3.5)
F	28 (2.2)
G	28 (2.2)
H	23 (1.8)
Other 59 small providers combined	114 (9.0)

The top four companies (Company A–D) operated kiosks in all six communes. The distribution was generally proportional to the overall distribution of kiosks by commune, with the exception of a few areas (e.g., Company B had a higher representation in Port-au-Prince commune). There was variable distribution of the smaller companies’ (Company E–H) kiosks by commune; for example, 95% of Company H kiosks were found in Delmas commune.

Kiosk operators were asked about water delivery mechanisms to the kiosk and kiosk equipment. Almost all (95.7%) reported that water was delivered via tanker trucks. Respondents at 35 kiosks (2.6%) stated that the water was provided on-site via a borehole, whereas 21 kiosks (1.6%) stated that water came directly from the piped water network. Among all kiosk respondents (*N* = 1,340), the majority (94.3%) reported that the water was filtered on-site before sale. Most kiosks had only a small cartridge filter for on-site treatment; however, 92 kiosks (6.9%) had membrane filtration units at the kiosk itself. The exact filter media type and specifications could not be verified, and knowledge of the filter type among respondents was low with more than half of respondents (63.3%) reporting they did not know the type of filter installed on-site.

Respondents were also asked about the water storage reservoirs on-site, in terms of specifications and cleaning practices. Of those who knew the reservoir size (*N* = 1,140), the majority (84.3%) reported having a reservoir between 1,000 and 2,000 gallons in size. The most common response was 1,200 gallons (51.0%), followed by 1,400 gallons (16.9%). For those kiosks where it was reported that the tank was cleaned (method not specified) (*N* = 1,272; 76.1%), the most common frequency was “every month” (25.7%), followed by “after each use” (21.7%). Sixteen percent of respondents did not know the frequency of cleaning.

### Sales volume and price.

**Figure 3.**
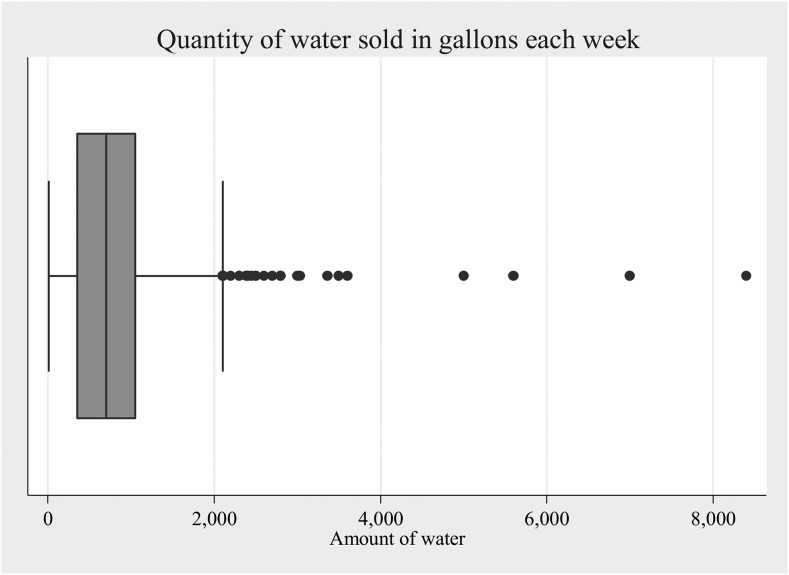
Box and whisker plot of reported weekly sales volume (gallons) for kiosks inventoried in metropolitan Port-au-Prince, July–August 2013.

All kiosks inventoried sold water by one and five gallon volumes. The reported total volume sold per week varied considerably. One-third of respondents (35.4%) did not know how much water they sold on a weekly basis. For those who reported knowing the volume sold (*N* = 845), the mean and median volume sold per week was 835.8 and 700 gallons, respectively. The highest weekly volume sold reported was 8,400 gallons (Figure 3).

The majority of kiosks (90.0%) sold water for 5 Gourdes (USD0.12 at the time of the inventory) per gallon; this equates to USD31.70 per m^3^. Using the mean volume sold per week, this equates to an average weekly gross sales per kiosk of USD97.41. If an individual were to purchase solely their drinking water from a private kiosk, this would equate to a weekly expenditure of on average USD0.67 on drinking water (annual total: USD34.69). Based on the per capita GNI in 2013, this would equate to spending approximately 4.3% of annual income on drinking water. If purchased water was used for basic consumption, this would equate to spending 10.8% of income annually.

### Kiosk inventory validation.

A total of 249 of the 278 kiosks with a supervisory visit (89.6%) were included in the validation process; kiosks lacking spatial information (two kiosks) and kiosks falling outside the reported segments (27 kiosks) were excluded. Almost all (93.6%) of the kiosks in the initial visits were also found in the supervisory visits. Over the entire area inventoried, this means that approximately 6.4% of kiosks (open, closed, or absent) or 85 kiosks may have been missed during the inventory.

### Provider company site visits.

Seven of the largest eight provider companies’ production sites were visited, including two separate production sites for Company A (eight site visits total; Figure 1). Company F was not visited because the owner could not be reached within the assessment period. Although not included in the questionnaire, it was reported during the visits that the top four provider companies have multiple production sites in metropolitan Port-au-Prince. Thus, the site visits comprised only a partial assessment of each company.

All eight sites visited reported having their own borehole on-site with the exception of Company H which stated that they use the piped water network as a water source. All providers reported RO membrane filtration as their main treatment mechanism; other processes including water softening, prefiltration with cartridge and carbon filters, and disinfection via ozone treatment and UV irradiation. Every company reported that they use their own trucks to deliver water to kiosks, with Company C reporting that they also use rental trucks to supplement their fleet.

### Water quality.

Free chlorine was not detected in any kiosk water sample from the 1,340 kiosks tested (all results less than 0.1 mg/L). Water samples from treated water at the providers also did not have detectable free chlorine residual; instead they reported use of UV and/or ozone for disinfection. The other physicochemical parameters tested from 767 kiosks (57.2%) and eight provider sites are summarized in Table 3.

**Table 3:** Physical water quality results from provider companies and kiosks inventoried in metropolitan Port-au-Prince, July–August 2013

		Provider sites (*N* = 8)* (raw water)		Provider sites (*N* = 8)* (treated water)		Kiosks (*N* = 767)	
Parameter	WHO target or acceptable range	Mean	Range	Mean	Range	Mean	Range
pH	6.5–8.5	7.0	6.8–7.3	6.0	5.5–6.75	6.4	5.3–7.9
TDS (mg/L)	< 600	573.7	322–915	34.6	8–104	22.5	2–268
Turbidity (NTU)	< 1	0.37	0.11–0.95	0.20	0.09–0.25	0.32	0.1–2.7

NTU = Nephelometric Turbidity Units; TDS = total dissolved solids; WHO = World Health Organization.

* Two provider sites were visited for Company A.

For the treated water samples from the providers, the parameters were within the WHO target or acceptable ranges, with the exception of pH. Six of the eight samples had pH below 6.5. Similarly, for the majority of the kiosk water samples, the treated water parameters were within the WHO target or acceptable ranges, with the exception of pH. Two-thirds (*N* = 512; 66.7%) of kiosk samples had pH below 6.5 (mean of 6.0); the remaining 255 samples (33.3%) were within the acceptable range. There were nine kiosks (1.2%) with turbidity outside of the target range. TDS measurements for all (100%) kiosk samples were within the target range.

There were 757 kiosk water samples analyzed at LNSP for *E. coli* (Table 4). All controls from the cooler boxes and laboratory met the WHO guideline value (< 1 MPN/100 mL) (for the negative controls) or were positive (for the positive controls) as expected.

**Table 4:** Microbiological (*Escherichia coli*) water quality results categorized according to WHO health-risk level for kiosk inventoried in metropolitan Port-au-Prince, July–August 2013

*E. coli* MPN per 100 mL (health-risk level)	No. of kiosks (%)
< 1 (conformity)	688 (90.9)
1–10 (low)	51 (6.7)
11–100 (intermediate)	17 (2.3)
> 100 (high)	1 (0.1)

MPN = most probable number.

Ninety-one percent (90.9%) of the samples met the WHO guideline value (< 1 MPN/100 mL). Of the 9.1% that tested positive for *E. coli*, the majority of these were in the WHO “low-risk” range (1–10 MPN/100 mL); however, 18 samples (26.0% of positives and 2.4% of all samples) were in the intermediate (11–100 MPN/100 mL) or high risk range (> 100 MPN/100 mL) (Table 4).

All of the kiosks that were positive for *E. coli* were resampled, and 58 of 69 (84.1%) met the WHO guideline value when tested a second time (15.9% were positive again). These second samples were collected an average of 11 days after the initial one (range: 2–25 days).

The proportion of samples analyzed for *E. coli* from each company was consistent with the overall proportions; however, the smaller providers are slightly overrepresented in the water quality results (Table 5).

**Table 5:** Total number of samples tested for *Escherichia coli* and percent positive for *E. coli* stratified by provider company during water quality assessments of kiosks in metropolitan Port-au-Prince, July–August 2013

Provider company	Number (%) of kiosks (*N* = 1,266)	Number (%) of total samples analyzed for *E. coli* (*N* = 757)	Number (%) of samples positive for *E. coli* by provider
A	588 (46.4)	340 (44.9)	32 (9.4)
B	218 (17.2)	115 (15.1)	11 (9.6)
C	150 (11.9)	87 (11.5)	3 (3.4)
D	72 (5.7)	40 (5.3)	3 (7.5)
E	45 (3.5)	29 (3.8)	1 (3.4)
F	28 (2.2)	17 (2.2)	7 (41.2)
G	28 (2.2)	15 (2.0)	4 (26.7)
H	23 (1.8)	7 (0.9)	1 (14.3)
Small providers	114 (9.0)	107 (14.1)	7 (6.5)

The *E. coli* results from each provider company were analyzed to determine if there were significant differences in the proportion of *E. coli* positive samples among the companies and also to determine if certain companies were contributing disproportionately to the *E. coli* positive results. Indeed, there were significant differences in the proportion of *E. coli* positive samples by provider company (*P* < 0.0001). Relative to the overall *E. coli* positive proportion of 9.1%, two companies were significantly different; Company C significantly lower proportion of *E. coli* positive kiosk samples (*P* = 0.01), whereas Company F had a significantly higher proportion of *E. coli* positive kiosk samples (*P* = 0.0075).

The *E. coli* results were also analyzed by delivery mechanism and treatment mechanism on-site to determine if there were associations with contamination. None (0%) of the 34 kiosk samples where the water was sourced from a borehole or the piped water network were positive for *E. coli*, compared with 69 of 723 samples (9.54%) of those that were delivered via tanker truck. For kiosk samples with RO units on-site (*N* = 49), the proportion of samples positive for *E. coli* was marginally lower than kiosks where RO units were not used at the kiosk level; 2.0% versus 9.6% (*P* = 0.07).

At the provider level, all treated water samples met the WHO guideline for *E. coli*; however, one raw water sample from a provider borehole was positive for *E. coli*. One to three trucks were also sampled for *E. coli* at six of the eight production sites visited (no trucks from Company C or Company G were sampled), with all samples (*N* = 12) meeting WHO guideline value.

## DISCUSSION

This inventory described both the scale and recent growth of the private kiosk sector in metropolitan Port-au-Prince. More than 1,300 private kiosks were identified in the six communes of metropolitan Port-au-Prince at the time of the inventory. Our inventory found that the water at more than 90% of the kiosks did not have detectable *E. coli* when sampled at the point of sale. Of those that had some level of *E. coli* contamination, most had concentrations less than 10 MPN/100 mL or in the low health risk level per WHO. However, there was variability in water quality by provider, with two companies having more than 25% of their kiosk samples contaminated. The resampling of contaminated kiosks suggested that contamination is likely periodic as opposed to systemic for most of the major companies and that approximately 10% of kiosks may be periodically contaminated somewhere between the site of production and the point of sale.

The use of decentralized, membrane-based, water refill stations is rapidly growing in other developing countries as well.^15^ In particular, Indonesia and the Philippines have seen explosive growth in businesses using similar technologies and business models.^15,16^ Sima and Elimelech^15^ report that the rate of growth of the sector in Indonesia was more than 800% over a 10-year period. Private, public–private, and nonprofit organizations, have also been introducing this type of water source to areas of India, Bangladesh, Ghana, Nigeria, Liberia, and Kenya.^17–20^ Despite acknowledgment of their increasing importance in urban water provision in developing countries, there is little documentation or research on these enterprises to-date.^21,22^ The sector in Haiti is one of the largest documented to-date, and it is in a minority category in that it is an entirely private sector venture and therefore largely demand driven.

In terms of pricing, use of private kiosks for drinking water only in Haiti would result in an expenditure of approximately 4% of annual income. The United Nations Development Program specifies an affordability threshold of no more than 3% of income on water.^19^ Therefore, this sector is potentially surpassing the affordability limit in Haiti.

Private-sector water, specifically sachet water, has been associated with both higher and lower quality water in several studies in developing countries; however, little evidence exists regarding the quality of water originating from membrane-based, refill stations.^23–25^ Proponents of membrane-based water treatment processes argue that this technology can provide high quality water, with complete removal of microbiological contaminants as well as chemical contaminants of emerging concern, such as heavy metals and pesticides.^26,27^ Sima and others^28^ found that use of this water type in Jakarta, Indonesia, was actually associated with lower diarrhea risk in children under 5 years. However, it was also separately documented that between 10% and 40% of stations sampled in the Jakarta study had some level of bacterial contamination.^15^ A limited evaluation of finished water from kiosks using membrane-based treatment processes in Ghana found that 91% of samples (*N* = 32) met WHO guidelines of nondetectable *E. coli* per 100-mL sample.^29^ Our inventory had similar findings to the limited literature available, with the majority of the kiosks assessed meeting WHO microbiological guidelines at the point of sale.

From the small number of treated water samples from provider sites or tankers, we did not detect *E. coli* contamination, however, this sample size was limited and therefore it is not possible to rule out potential contamination at these stages of the distribution process. At the kiosk level, reported tank cleaning practices were variable. Although the majority (76%) indicated the tank was cleaned periodically, there was a lack of standardization of procedures. This, and other variables in kiosk equipment type, may be contributing to periodic contamination of a small proportion of private kiosks.

There is also potential for contamination at the household level, given the lack of chlorine residual protection in this water and the well-documented phenomenon of secondary contamination during transport or unsafe storage in the home.^30^ A survey from early in the cholera outbreak suggested that people had more confidence in the safety of this water relative to other sources^5^; therefore, they may be less likely to use forms of household water treatment such as chlorine tablets, especially given the price paid for this already treated water.

From a regulatory perspective, this inventory allowed an estimation of scope of the private kiosk sector in metropolitan Port-au-Prince in 2013. It also highlighted the challenges to implementing government oversight given the rapid growth of the sector. Although the number of providers recorded was large, the majority of kiosks inventoried were franchises of only a handful of large provider companies. Further, the majority of kiosks reported similar water delivery, treatment processes, configuration, and pricing structure. Taken together, this may facilitate development and dissemination of standardized operating procedures (SOPs) in the short term, in addition to longer-term implementation of certification courses, as have been implemented in the well-developed sector in the Philippines.^16^ SOPs and kiosk operator training should reduce the likelihood of contamination at the point of sale; however, the enforcement of water quality guidelines and independent routine monitoring of water quality will be important to ensure public safety. Implementation of effective monitoring schemes and enforcement by local health offices has proved challenging in practice.^15,16^

The results from this inventory are subject to several important limitations. Because of time and resource constraints, this baseline inventory only represents a portion of metropolitan Port-au-Prince. We cannot extrapolate these findings to the remaining, uncovered areas, as the density of kiosks may have varied and the providers in these uninventoried areas may have been different. We also did not ask on our questionnaire how many of these kiosks inventoried were present prior to the earthquake (i.e., were destroyed and then rebuilt), therefore, we cannot determine the rate of growth of the sector. Furthermore, we cannot extrapolate these findings to other cities in Haiti. Next, due to shortcomings of the methodology and/or errors in field procedures, a small proportion of kiosks were missed within the areas covered by the inventory. Next, much of the data collected was reported, not observed, (e.g., frequency of cleaning and equipment type), and may not accurately represent the situation at the kiosk level or at the major providers. Additionally, while all agents received the same training and oversight, certain field agents were more astute at probing and interpreting the answers to questions from the questionnaire. The microbiological results only provide a snapshot of water quality at one point in time. Longitudinal sampling is required to understand the overall microbiological safety of water sold from private kiosks, the levels of systemic versus periodic contamination, and the quality of water originating from specific providers. Finally, the major provider inspections were not exhaustive and therefore cannot be used to make generalizations about each provider nor the tanker truck distribution network.

## CONCLUSIONS AND RECOMMENDATIONS

In conclusion, this inventory in 2013 represented the first, comprehensive assessment of the scale and characterization of the private water kiosk sector in metropolitan Port-au-Prince. Further, it was a major initiative by the Government of Haiti to move toward regulation of private sector drinking water providers. In the context of post-emergency in Port-au-Prince, with underlying challenges of basic service provision due to rapidly growing and unplanned urbanization, it is clear that the private sector is contributing significantly to meeting the drinking water needs of the population. The scale of this membrane-based, refill kiosk sector in Port-au-Prince represents one of the largest documented in the literature to-date, highlighting the importance of regulation. Although the national government works to increase access to piped water, private kiosks will undoubtedly continue to play a role both in Port-au-Prince and potentially in other peri-urban and even rural areas of Haiti.

Additional study at the household level, in terms of water source perceptions, practices, and preferences may help develop additional guidance for regulatory purposes, as well as to understand the extent of use of this kiosk water relative to affordability and access to other source types. It is recommended that SOPs are developed in the short term along with implementation of training in the longer term to ensure microbiological water quality up to the point of sale. Finally, in the context of ongoing cholera flare-ups and high rates of diarrheal disease, it is recommended that the government continue with public health messages regarding the importance of household water treatment and safe storage, regardless of source type, as this will ensure that drinking water is safe at the point-of-consumption.
